# The emergence of family medicine in India–A qualitative descriptive study

**DOI:** 10.1371/journal.pgph.0001848

**Published:** 2023-05-12

**Authors:** Archna Gupta, Ramakrishna Prasad, Sunil Abraham, Nisanth Menon Nedungalaparambil, Onil Bhattacharyya, Megan Landes, Sanjeev Sridharan, Carolyn Steele Gray

**Affiliations:** 1 Department of Family and Community Medicine, St. Michaels Hospital, Toronto, Canada; 2 Department of Family and Community Medicine, University of Toronto, Toronto, Canada; 3 Institute of Health, Policy, Management and Evaluation, University of Toronto, Toronto, Canada; 4 PMCH Restore Health, Bangalore, Karnataka, India; 5 National Centre for Primary Care Research & Policy, Academy of Family Physicians of India (AFPI), Ghaziabad, India; 6 Department of Family Medicine, Christian Medical College, Vellore, Tamil Nadu, India; 7 Department of Emergency Medicine, AIMS, Thrissur, Kerala, India; 8 Health Policy Evaluation, Social Science Research Institute, University of Hawaii at Manoa, Honolulu, Hawaii, United States of America; 9 Bridgepoint Collaboratory for Research and Innovation, Lunenfeld-Tanenbaum Research Institute, Sinai Health System, Toronto, ON, Canada; Indian School of Business, INDIA

## Abstract

Countries globally are introducing family medicine to strengthen primary health care; however, for many, that process has been slow. Understanding the implementation of family medicine in a national context is complex but critical to uncovering what worked, the challenges faced, and how the process can be improved. This study explores how family medicine was implemented in India and how early cohort family physicians supported the field’s emergence. In this qualitative descriptive study, we interviewed twenty family physicians who were among the first in India and recognized as pioneers. We used Rogers’s Diffusion of Innovation Theory to describe and understand the roles of family physicians, as innovators and early adopters, in the process of implementation. Greenhalgh’s Model of Diffusion in Service Organizations is applied to identify barriers and enablers to family medicine implementation. This research identifies multiple mechanisms by which pioneering family physicians supported the implementation of family medicine in India. They were innovators who developed the first family medicine training programs. They were early adopters willing to enter a new field and support spread as educators and mentors for future cohorts of family physicians. They were champions who developed professional organizations to bring together family physicians to learn from one another. They were advocates who pushed the medical community, governments, and policymakers to recognize family medicine’s role in healthcare. Facilitators for implementation included the supportive environment of academic institutions and the development of family medicine professional organizations. Barriers to implementation included the lack of government support and awareness of the field by society, and tension with subspecialties. In India, the implementation of family medicine has primarily occurred through pioneering family physicians and supportive educational institutions. For family medicine to continue to grow and have the intended impacts on primary care, government and policymaker support are needed.

## Introduction

### Family medicine and strengthening PHC

Countries globally seek to establish well-functioning primary health care (PHC) systems to meet the health needs of their populations. PHC addresses the majority of a person’s health needs throughout their lifetime [1]. This includes physical, mental and social well-being and is people-centred rather than disease-centred [1]. There are multiple paths to strengthening PHC. PHC requires high-quality and safe primary care (services delivered to individuals), essential public health functions (services delivered to populations), multisectoral policies and action, and empowered people and communities [2]. Leveraging all four areas of PHC is ideal, but countries may choose to focus on some or none. In some instances, countries focus on strengthening primary care as the path to supporting PHC [3]. One way of achieving this is through developing and implementing family medicine (FM).

Primary care is defined as "first-contact, continuous, comprehensive, and coordinated care provided to populations undifferentiated by gender, disease, or organ system" [4]. The four main features of primary care services are first-contact access for each new need, long-term person- (not disease) focused care, comprehensive care for most health needs, and coordinated care when it must be sought elsewhere. Based on the landmark research by Barbara Starfield, the global community judges primary care as "good," according to how well these four features are fulfilled [5]. In some countries, primary care functions are distributed among a group of specialist doctors, with pediatricians caring for children, internal medicine physicians caring for adults, and obstetricians caring for pregnant women. Other countries have taken a different approach to primary care delivery by family physicians who are trained to provide care for the entire community [6].

FM is a subset of primary care delivered by family physicians—specialists in primary care [3]. Family physicians are differentiated from general practitioners (GPs) by completing postgraduate training in family medicine (FM), while GPs entered practice after completing medical school [6]. Globally, the terminology varies, with the terms "general practitioner" and "family physician" used interchangeably. In some cases, the term GP does include those who have specialized postgraduate training in primary care (as seen in the UK, Denmark, Netherlands and Australia, for example). While in other contexts, it refers to a physician who has completed an undergraduate medical degree with no specialty training and provides generalized medical care at a grassroots level [6, 7]. In this research, we use the term "family physician" to identify those that have completed postgraduate training in FM [3].

FM training follows eight core principles that guide its education and training: access or first-contact care, comprehensiveness, continuity of care, coordination, prevention, family orientation, community orientation, patient-centeredness. These principles build on those aligned with good primary care (first-contact, continuous, comprehensive and coordinated), adding the components of prevention, family orientation, community orientation and patient-centeredness. These eight principles distinguish FM from other medical specialties and other primary care providers [6].

FM is context-specific; "the scope of each family doctor’s training and practice varies according to the contexts of their work, their roles, and the organization and resources of the health systems in each country" [6]. There is not a "one size fits all model" for FM practice. For example, in low and middle income countries (LMIC), and remote regions of high income countries, with few medical practitioners, family physicians may work in secondary care settings performing surgical procedures, including cesarean sections, managing trauma and care for adults and children. In other contexts, family physicians may be a clinical lead for a hospital-based healthcare team or have leadership or administrative roles within teams [8].

Family physicians exercise their professional role by providing care directly to patients or working collaboratively with other health professionals based on the health needs and resources available in the communities they work [6, 9]. Primary care can be delivered under different models. In a FM model, family physicians are trained along eight principles which allows them to have diverse professional roles and includes skills that allows them to follow patients through their life course, to work across primary and secondary care sectors, and act as gatekeepers to specialized care [3, 6]. It is the breadth of their roles and skills which allows them to meet the primary care needs of their communities. Having family physicians deliver and manage primary care increases the scope of care provided to patients and improves the efficiency and organization of primary care delivery [10–12].

In LMIC, where there is not a critical mass of physicians, much of first-contact primary care is provided by non-physician providers (nurses, community health workers, etc.). Despite this, we see the introduction of FM over time in many countries as health systems evolve. In the 1980s, we saw postgraduate training in FM initiation in Brazil, Chile, Venezuela, Lebanon, Sri Lanka, Nepal, Thailand, Uganda, and Nigeria [13–21]. In the 1990s in Uruguay, Turkey, India, and Vietnam [13, 22–24]. Since 2000, many Sub-Saharan Africa countries have implemented FM training, including Ghana, Botswana, Kenya, Nigeria, Lesotho, Ethiopia, Malawi, and Zimbabwe [6, 7, 25]. However, many countries have not made much progress in their implementation process, meaning FM is not an integral part of primary care delivery in these countries.

India is one such example that has been undergoing FM implementation since the 1980s through advancing training opportunities to standardize and improve the skills of primary care providers with a focus on improving quality and access to care across different regions. While implementation progress has been slow and gaps remain, the advancements must be understood. This paper seeks to uncover how early cohort family physicians–those willing to enter a new field in their context—have shaped the implementation of FM in India.

### The evolution of India’s primary health care system

It is helpful to know how India’s PHC system has evolved to understand its gaps today. In 1946, post-independence, the Bhore Committee report outlined India’s intention to have a universal health system. This tax-funded national health system was meant to deliver care through a three-tiered public system [26]. However, faced with several challenges, including partition, poverty, and a weak economy, India’s universal health system was never realized. Although much progress has been made over the last 70 years, India’s healthcare system remains one of the most fragmented and unequal globally.

Before independence, there were two classes of physicians, those who undertook 5.5 years of training and obtained medical degrees and those who carried out shorter training programs and were considered licentiate medical practitioners [27]. In addition to physicians, there were many traditional health providers, including Ayurveda, Yoga and naturopathy, Unani, Siddha, and Homoeopathy (AYUSH collectively) and untrained medical providers. At the time, two-thirds of medical practitioners were licentiates who predominantly worked in rural areas. A part of the Bhore committee recommendations was a vision for standardized and high-quality care, which ended the regulated system for licentiate medical practitioners with shorter training and excluded AYUSH providers [27, 28]. The intention was that all medical doctors would minimally be qualified as GPs, meaning they have an undergraduate medical degree—Bachelor of Medicine and a Bachelor of Surgery (MBBS)—which is equivalent to an undergraduate MD degree in the US or Canada.

Although the recommendations intended to ensure high quality of care for patients, what resulted was a system that had a severe shortage of trained and regulated health professionals in both the public and the private healthcare systems. India was unable to produce the number of GPs needed. With population growth and epidemiological shifts, the population’s needs continued to rise. The result was a vast market for unregulated and untrained healthcare workers to “set up shop” in the private sector to meet societal demands. In 2014, the private sector accounted for 75% of all primary care visits and 70% of all health expenditures [28–30]. In rural India, 86% of primary care providers work in the private sector, and 68% have no formal training and are operating outside the discretion of health policy and regulation [28].

In the past decade, multiple initiatives have been employed to improve the severe shortages in primary care human resources and support strengthening PHC. First, in 2014, realizing that India could not produce enough GPs to meet the population’s needs, the federal government standardized AYUSH providers, allowing training and recognition with oversight through an established Ministry of AYUSH [28]. Second, the number of medical colleges in the country expanded, increasing from approximately 200 in the early 2000s to over 400 in 2016 [31]. Despite greater medical school training opportunities, most medical graduates pursue a postgraduate specialization instead of working as GPs in primary care, particularly in public and rural sectors [32]. Further compounding the issue is the concern that graduating GPs are not competent to provide high-quality primary care to the population given variable undergraduate medical education between schools [27, 28, 33].

In 2018, the Government of India introduced Ayushman Bharat (AB), which has two aims. First, it includes a national health insurance program, Pradhan Mantri Jan Arogya Yojana (PMJAY), which covers secondary and tertiary level care services at public and private hospitals for India’s poorest 40% of the population. Second, it establishes 150,000 government-owned Health and Wellness Centres (HWC) [34, 35]. The HWCs are not new centres but are revitalized existing subcentres that have historically been understaffed or under-resourced and consequently closed or underutilized because a lack of access to quality care [27, 36].

India’s primary care sector is three-tiered and comprises subcenters (now being upgraded to HWCs), primary health centres and community health centres (CHCs) [37]. Primary health centers are the first contact between a community and a medical officer, who may be an MBBS graduate or an AYUSH provider [38]. CHCs are referral sites to four primary health centers, which require four medical specialists, including an internal medicine physician, an obstetrician-gynecologist, a pediatrician, and a surgeon, to be operational [39]. However, finding specialists to work in these centres is an immense challenge where over 50% of specialist positions in CHCs are vacant, resulting in many being closed [27].

In this model, which began implementation in March 2019, HWCs are promoted as being upgraded by governments to provide “comprehensive PHC services” [40]. In the HWCs, care is intended to be delivered in a team model by mid-level healthcare providers or community health officers with six months of training, multi-purpose female workers, and AYUSH providers led by a non-physician team leader [41]. This newly proposed non-physician model in AB’s HWCs is meant to resolve the existing system’s over-reliance on physicians. In this model, healthcare workers at HWCs are intended to receive support through referral to primary health centers, community health centers, or teleconsultation [42]. Although the changes made through the AB reforms, specifically the implementation of HWCs, are essential, questions remain unanswered. For example, how will ongoing training and mentorship of mid-level healthcare providers be ensured, and how will the lack of skilled specialist physicians in CHCs be resolved.

### The need for family medicine in India

India is a valuable case example where we can explore the implementation of FM via the institutionalization of training to strengthen PHC. In the era of specialty care, FM is more critical than ever in India to provide a cadre of specialist physicians equipped to deliver primary care and work with and increase the capacity of existing traditional, low-level, and perhaps informal providers. FM can only gain momentum once there is a sufficient supply of family physicians to deliver services, highlighting the critical role of innovators in designing and implementing FM training and early adopters’ willingness to enter this new field. This research explores how advancing family medicine training through the work of early cohort family physicians has influenced the implementation of FM in India.

### Implementation

Implementation is the process of putting to use or integrating new practices within a setting [43–45]. In this research, we are concerned with implementation that leads to institutionalization, whereby FM becomes routinely embedded and normalized into the Indian context [43–45]. One way in which institutionalization can be achieved is through the establishment of formalized training, which gives rise to new roles and processes [46]. Given the unique implementation environment of India, which was driven in part by advancing training opportunities, this study explores the role of innovators and early adopters–collectively viewed as pioneers—in the implementation of FM in India, focusing on the role of training. Training is an important area of focus as the health workforce is a crucial contributor to people-centred primary care. There has been insufficient investment in training primary care staff in the past forty years, which countries are attempting to address via an investment in training [47].

This study seeks, in particular, to understand the role of individuals and leaders in driving the implementation process. Roger’s classical theory, the “Diffusion of Innovation’s theory,” explains how innovation ideas spread among individuals, particularly harnessed by the influence of leaders and change agents [48]. Specifically, this theory helps explain the role of “innovators” and “early adopters” in the implementation process [48]. Accordingly, we focus on early cohort family physicians and FM training program implementers because of their willingness and motivation to enter a novel field and their critical role in forging new paths for FM to emerge through their work. Greenhalgh et al.’s Conceptual and Theoretical Bases for the Spread of Innovations can be used to build on Rogers’ foundational work to help understand how innovators and early adopters support the spread of innovations, in our case FM training [45]. This determinant model suggests several domains that act as enablers or barriers that influence implementation in complex situations, appropriate for understanding FM implementation in a regional context. We focus on the following model factors: adoption by individuals, communication, organizational contexts, health system readiness, and the outer context, including policy-level changes. We do not specifically use either Rogers’ or Greenhalgh’s models to describe the innovation, including the various FM training programs. The focus of this study was the large-scale implementation of training leading to the field’s professionalization as opposed to individual training programs.

### Aim

This study had two broad aims: (1) to understand how FM is implemented nationally and (2) to understand how FM implementation influences PHC system strengthening. To address these aims, we had two research questions: (1) what are the potential mechanisms by which early cohort family physicians drive and sustain FM implementation in India and over what trajectory? and; (2) what are the mechanisms by which early cohort family physicians strengthen PHC in India?

Given that there were two specific research questions and the volume of data, this study is reported in two articles. This article addresses the first research question, describing how early cohort family physicians supported the emergence and implementation of FM in India. A second article will describe how early cohort family physicians support strengthening PHC in India.

## Materials and methods

### Ethics

This study was reviewed and accepted by the Research Ethics Board at the University of Toronto, Canada and from the Institutional Review Board (Health) at the Swami Vivekananda Youth Movement, India. Written consent was obtained from participants before participation in interviews. Consistent with participatory approaches, participants were given a choice to be identified by name or anonymously, enabling and empowering them to retain ownership of their stories [49].

### Study design

A participatory action research approach which applies qualitative description (QD) collection and analysis methods was used [50, 51]. The research questions were born from discussions with Indian family physician collaborators who challenged that one could not understand the emergence of FM in India without hearing the pioneers’ stories. As one of our collaborators put it: “If you do not listen to these stories, you will miss out a lot on the pioneering work for FM that happened in India”(SA). Indian family physician collaborators (RK, SA, NM) were involved in the research design, including defining the questions, designing the research protocol, and participating as research participants (SA). Action research engages professional social researchers with local stakeholders in a co-generative process of knowledge creation, action design, and outcomes evaluation [51]. Participation in action research is not just a “moral value” but essential because of the complexities of the problems addressed, ensuring participant knowledge is used to inform research practice [50].

Applying a QD collection and analysis approach was appropriate for this type of exploratory inquiry in which there is limited pre-existing research. QD is also appropriate to help describe and understand the underlying processes of FM implementation and PHC strengthening from the perspectives of participants [52–55]. In QD, researchers are actively involved in the research process where their understanding of the phenomena, in this case, FM, are seen as integral for new inquiry [52, 56, 57]. This was important for our study given that several authors involved in this study are family physicians themselves (AG, RK, SA, NM, OB, ML) and in the Indian context (RK, SA, NM). Using QD also allowed us to remain faithful to the participants’ accounts of FM emergence [57, 58].

### Participant recruitment

We identified FM pioneers in Tamil Nadu, Karnataka and Kerala, including early cohort family physicians, who received the designation through the MNAMS certification route or completed a three year full-time Diplomate of the National Board (DNB) postgraduate training in Family Medicine, or were teachers of the early cohort family physicians. These states were selected as they were the first to have introduced postgraduate training in FM and have the greatest number of training programs. Initially, purposeful sampling was used to identify participants known to have contributed to the field of FM [59, 60]. Participants were identified by Indian family physician collaborators and their networks. As the study progressed, snowball sampling was used, by asking participants to suggest others they would recommend for this study at the end of each interview[59].

### Data collection

To answer our research questions, we aimed to interview fifteen to twenty participants to allow for information-rich and detailed cases permitting thick descriptions [52, 61, 62]. Given that QD analysis emphasizes each participant’s individual experience and story’s uniqueness, data saturation can never truly be achieved and was not used as a parameter for determining sample size [52, 63]. We focused on a size that allowed us to adequately explore and answer the research question [52].

In-depth interviews were conducted. A semi-structured interview guide was used. Interview guides were pilot tested before their use with two Indian family physician research collaborators (RP, NM) to ensure question clarity and reduce redundancy [64]. Interviews were conducted in English by the principal author (AG) and were audio-recorded and transcribed verbatim. Interviews ranged from 40 minutes to 75 minutes. All interviews took place between August and October 2019 with one to two interviews daily.

Supplementary field notes and reflective memos were written after each interview to capture observations about the participants’ settings and geographic context, allowing the interviewer (AG) to immerse herself in the environment and to put participant stories into context, understanding how, where and to whom participants delivered care [65, 66]. Reflective memos allowed the interviewer (AG) to document reflections and perspectives on individual interviews, create a synopsis of each conversation, and note the data’s interpretation throughout the process, remaining attentive to what was unique or common across interviews.

### Data analysis

Interview field notes and memos were reviewed after each interview (AG). Researchers (AG and RP) discussed emerging themes throughout the three-month interview process, allowing early insights to be incorporated into ongoing data collection [66]. Transcription of interviews was done in two sets, first after interviews that were conducted in Tamil Nadu and then again after interviews were completed in Karnataka and Kerala. “Strategic periods of immersion in the field” occurred when the interviewer (AG) was actively interviewing participants in Southern India. This was “interspersed with periods of immersion in the data,” which supported testing and developing conceptualizations about the roles that early pioneers played in defining and implementing FM in India [67]. After repeated immersion in the data and “making sense of the data,” the transcripts were coded to identify categories, themes and linkage in the data and explore relationships and patterns between the data sources [52, 57]. Iterative, inductive analysis techniques were applied. Inductively identified codes were mapped onto the implementation frameworks described earlier.

A sample of six interviews was read, re-read and hand-coded to develop a codebook with emerging themes and subthemes [52]. The codebook was validated with a peer researcher. AG and the peer researcher independently read and coded three diverse transcripts (state of training, gender of participant, type of work) and compared findings to ensure consensus on the definition of codes. The codebook and a summary of the initial findings were sent to all participants for feedback. We received a response from 50% of the participants interviewed and no changes were made to the codebook. After hearing back from participants, all twenty transcripts were then re-read and hand coded. Analytic notes were taken during this process to document emerging connections and patterns within and between themes. All interviews were coded again, allowing for further reflection and organization of codes and for digitization of hand codes in NVivo 12 Plus [68].

## Results

### Participants

Twenty-two participants were invited to participate. Twenty accepted (91%), one declined, and one did not respond. Participant characteristics are described in Table 1. Ten participants were identified by purposeful sampling alone (50%), three were identified by snowball sampling alone (15%), and seven were identified by both purposeful and snowball sampling (35%). Eighteen interviews were conducted in the setting where participants work. Two interviews were completed via Zoom due to scheduling conflicts. Of the twenty participants, nineteen (95%) participants chose to be identified by name, and one opted to be anonymous.

**Table 1:** Participant characteristics.

Current State of Practice*	
Tamil Nadu	8
Karnataka	6
Kerala	6
**Family Medicine Certification**	
MNAMS-FM	3
International FM Degree	1
DNB-FM	15
None	1
Current Location of Practice**	
Rural	7
Urban	6
Academic	7
**Gender**	
Female	8
Male	12

*State of practice does not necessarily reflect the state of training.

**Some participants had exposure to multiple settings and were included under the setting that they practiced at the time of the interview.

### Innovators and early adopters

Rogers’ Diffusion of Innovation theory helps us understand the roles of individuals involved in implementation. Greenhalgh Conceptual and Theoretical Bases for the Spread of Innovations help us to understand the motivations and actions of innovators and early adopters in enacting those roles. Innovators recognize the need for change, are willing to take risks, and develop and implement new ideas [48]. Early adopters embrace opportunities, are aware of the need to change, and are thus comfortable adopting new ideas and leading change [48]. Innovators are seen as the first to implement FM training programs. The first cohorts of FM trainees are considered early adopters. From our sample three participants were identified as innovators and 17 as early adopters. Early adopters were willing to seek out innovations, experiment with them, find meaning in them, gain experience, and even modify them [45].

In this section we break down the motivation and activities of innovators and early adopters to illustrate their roles in the implementation process. Motivations stemmed from recognizing the need and desire to increase the scope of skills of physicians working in the primary care sector to better serve patients and communities. This led to individuals starting the first FM training programs and others to be the first learners to enrol in this new field of medicine in the Indian context.

#### Motivations of innovators [Greenhalgh]

Innovators shared different motivations in their drive to implement various types of FM training programs–ranging from informal sessions to certification programs to formalized postgraduate training programs. All three innovators recognized a need to improve the skills of general practitioners (GPs), which motivated them to be involved in the implementation of various types of training programs in different States and regions over time. These realizations stemmed from personal experiences regarding limitations in their own training or from specialists recognizing the gaps in the availability of primary care in the communities they served.

In the early 1980s, before the formal implementation of postgraduate FM training programs, some GPs attained certification in FM by passing a written and practical examination and were referred to as Members of the National Academy of Medical Sciences (MNAMS)–Family Medicine. Participants practicing as family physicians in this decade shared feeling “disenchanted” and “frustrated” that they were not ready to manage the complex needs of patients following their MBBS training. They described wanting to find a way to share practice experiences and update their knowledge on an ongoing basis to meet their patients’ needs. In Karnataka, Dr. BC Rao initiated a professional organization for family physicians to come together and learn from one another in 1981 that continues to meet today (Table 2, Quote 1).

**Table 2:** Innovator motivation quotes.

Quote Number	Quote	Participant
1	“Some of us thought it is time that we started an organization of our own to look after the education and interests of doctors who are in FM practice and that’s how the Physicians Association—in those days we used to call it as IAGP, Indian Association of General Practitioners—came into being…then I made a smaller group which could share the practice experiences and then update, I felt the requirement of this kind of neighbourhood practice, so I started a club in my area with three members, and again it grew to five, then ten, and then it became fifteen, always these meetings were held in the homes of doctors.”	Dr. B.C. Rao, Karanataka
2	“I was covering General Medicine, but I realized having worked for a short time that the more vulnerable people around the community were not accessing healthcare despite it being of lower cost. They were accessing healthcare often in a delayed or not at all, and therefore the best way to try and care for those sections of the community is through FM. That is why I chose to do FM, and I felt that one of us is supposed to train people in that specialty of FM.”	Dr. Rajkumar Ramasamy, Tamil Nadu
3	“One of the pediatric surgeons graduated from CMC, Dr. Vinod Shah. He was managing 25 mission hospitals in an organization called Emmanuel Hospital Association…He realized that even though he was managing complex problems in the mission hospitals, the root of the problems were not managed well in primary care. So, he realized that he should develop a course for teaching the existing general practitioners.”	Dr. Venkatesh S., Tamil Nadu

In 1986, Dr. Rajkumar returned to work in a small, private, rural mission hospital, Christian Fellowship Hospital (CFH) in Oddanchatram, Tamil Nadu, after completing internal medicine training in the United Kingdom (UK). After working a short period, he realized a significant gap in primary care in the community he was serving. Given his experience with established family physicians in the UK, he realized this was missing in India (Table 2, Quote 2). In 1989 he implemented a “rural medicine rotation” focusing on increasing the capacity to deliver primary care to rural communities. He then formalized this program in 1991 into the first Diplomate of National Board in Family Medicine (DNB-FM) program, accredited by the National Board of Examinations (NBE), after stumbling upon the fact that FM was listed as Medical College of India (MCI) specialty as of 1984.

In 2006, a pediatric surgeon who spent decades working in and managing mission hospitals in various states identified a need for GPs in the country to be re-trained to better serve the needs of communities (Table 2, Quote 3). He developed one of the first and largest blended-distance education programs in FM at the Christian Medical College (CMC) in Vellore, leading to a Post Graduate Diploma in Family Medicine (PGDFM). Part-time, blended FM programs are currently not accredited and do not confer certification by the medical council of India in FM after completion.

In Kerala, Dr. PK Sasidharan was interested in community-oriented primary care when he started to practice medicine but had “no idea about FM” in India. He learned about FM and its impact through exposure to other countries. In parallel, he began to see the development of DNB-FM programs. He and other critical innovators in Kerala recognized and advocated that FM needed to be present nationally in public government medical colleges for successful implementation and buy-in of the field. Historically, these institutions have been perceived as the gold standard for India’s medical education. In 2007 the Calicut Medical College submitted a request to the Kerala government to start an MD-FM program. The university received approval from the Kerala government to start the program five years later, in 2012, making it the first MD-FM program in India.

#### Motivations of early adopters [Greenhalgh]

Early adopter participants shared about their motivations to train in FM and pursue a profession in a historically neglected and undervalued sector. This is particularly noteworthy as this occurred at a time when physicians were increasingly moving towards working in subspeciality sectors in secondary and tertiary care. Some individuals in this group sought out a field like FM before they knew it existed, as it resonated with their motivation and values for entering the field of medicine in general (Table 3, Quote 4). For others, FM training met their specific needs to be generalists in rural or remote regions where the skills they learned during their MBBS training alone would be insufficient (Table 3, Quotes 5 and 6). Others worked in primary care as GPs after completing their MBBS alone before pursuing postgraduate training in FM. During this time, they realized their desire to be generalists. They felt ill-prepared to serve the population without additional skills (Table 3, Quote 7, 8, 9).

**Table 3:** Early adopter motivation quotes.

Quote Number	Quote	Participant
Motivations		
4	“My dad was always reminding me of the necessities in remote areas … the poverty, which is there in the villages, lack of health awareness, lack of literacy. So that put an interest in me that whatever branch I take, I need to help the rural poor. So that was the main interest, and I felt that FM addresses this need.”	Dr. Isac David, Tamil Nadu
5	“I had always aimed to go to a place where other doctors were not willing to go, so I figured that I may be the only doctor. So, I would need to know about how to manage many areas of medicine.”	Dr. Vijila Isac, Tamil Nadu
6	“I started practicing here because this is my native place. So, I want to serve my people, and that is the reason I did my FM”	Dr. Bhaskara Puttarajanna, Karnataka
7	“I practiced as a GP for three years. So, I can say I learned a lot of patients’ contexts during that three years, patients’ needs, which are very different from what is taught in a traditional [undergraduate] medical school curriculum.”	Dr. Venkatesh S., Tamil Nadu
8	“For me, the initial motivation to become a family physician is because I wanted to work in a place where I am relevant for the country and in that place, I needed to be multi-skilled. So, at that time, I did not know anything about the principles of FM; I just wanted to be multidisciplined.”	Dr. Jachin Velavan, Tamil Nadu
9	“After my medicine [MBBS], I went and worked in a rural mission hospital in Central India. This was a 45-bedded hospital where I was the only doctor for some time, but I had to manage patients and everything, busy hospital, had to do deliveries and manage children and everything, minor surgeries, fractures… Then I realized that India needs more general physicians, that the need of rural India, it is so different from Kerala, where I come from. I realized that you know you can’t expect multiple specialists to work here, and I realized the need for a general physician. Then I went and worked in Christian Fellowship Hospital, Oddanchatram… they had started the new DNB training in FM, and I felt more and more convinced this is what is needed.”	Dr. Sunil Abraham, Tamil Nadu

Understanding the motivations behind innovators’ and early adopters’ willingness to deviate from the norm is important. From these examples, we see how both innovators and early adopters saw a need to increase the capacity of physicians working in the primary care sector. Specifically, MBBS training alone and thereby GPs in the Indian context require additional skills and training to meet the needs of communities.

#### Activities of innovators and early adopters [Rogers and Greenhalgh]

In Greenhalgh’s model, interventions may be implemented through several different mechanisms along a continuum [45]. On one end, there is “passive diffusion,” a “let it happen” or a “bottom-up” approach where innovations are implemented in an unpredictable and emergent way [45]. In the middle implementation is facilitated through a “help it happen” process with external agents supporting or influencing the change [45]. Finally, on the far end, there is “active dissemination,” a “make it happen” or “top-down” approach, which is highly planned and supported [45]. In this research we found many examples of how FM was implemented in an emergent fashion through the actions of innovators and early adopters. They developed training programs, supported implementation in multiple training institutions, taught and mentored the first cohorts of family physicians, and advocated for the field’s growth.

From the last section, we saw that many innovators implemented the first training programs. They were also the teachers of the first cohorts of family medicine trainees. Those early cohort family physicians were then involved with developing and implementing FM training programs in other institutions, promoting the spread from one institution to others. Individuals in these early cohorts are best understood as early adopters, who then took on some of the activities and roles of innovators to continue to proliferate training to others.

FM training continued to spread as early adopters started advocating for FM and creating more programs across the country. They are seen as “champions” who support the diffusion of innovations within organizations. Dr. Jachin, trained by innovators at CFH, speaks about how she now trains family physicians through the part-time, blended PGDFM program (Table 4, Quote 10). Dr. Sunil, also taught by innovators at CFH, speaks about developing faculty training programs to support and mentor family physicians in implementing new FM training programs in other institutions to help further spread (Table 4, Quote 11).

**Table 4:** Innovators and early adopters activities.

Quote Number	Quote	Participant
10	“Those we have trained are making changes but in pockets across the country and India is a 1.3 billion population country and 2000–3000 [family physicians] is like a drop in the ocean. But what has happened is because of that, the voices of family physicians has started being heard … Parallelly what Dr. Sunil and others have done like MD in FM or pushing for that … so they are now coming to the table to talk about with you so we can increase the talk, so it is a movement, it is slow… it is not easy initially to establish this specialty, so we are in that state… we are in a very much in an advocacy stage where we are showing that FM can be helpful.”	Dr. Jachin Velavan, Tamil Nadu
11	“I am trying to get some people whom I know who can teach and have similar [FM program development] training hubs around the country where people can come once in three months, learn from each other, and go. So, I think we have to be proactive. They have to do it because we cannot wait for people to come and teach.”	Dr. Sunil Abraham, Tamil Nadu
12	“Dr. Raman Kumar. He is one of the early members who wanted to have an academy and wanted to start this movement of Academy of Family Physicians of India, and they have been able to do so much influence on the policymakers also, and they are gaining footholds into medical colleges wherein they orient the students right from their initial years into FM.”	Anonymous
13	“After ten years, we think that now the new curriculum has come in MBBS, they have initiated a foundation course of one month and in that there is one hour dedicated for principles of family practice in MBBS undergraduate curriculum nationally… I think that one hour should be made into at least one month posting with rotation for students in MBBS.”	Dr. Resmi S. Kaimal, Kerala

Early cohort family physicians have also taken on the roles of leaders and advocates to help drive implementation. “Transformational leaders” are individuals who harness support from their organizations to promote change. These roles align with Greenhalgh’s model’s “change agents” who also support innovation diffusion through developing relationships, empowering early adopters and advocacy with external agents [45]. Dr. Raman Kumar, who launched the Academy of Family Physicians of India (AFPI) in 2010, is one such example. Many of the early adopters interviewed in this study are also members of AFPI or past presidents of state chapters. The AFPI has brought together family physicians from across states and nationally, creating a space for networking and advocacy (Table 4, Quote 12). The AFPI has advocated for policy change at a national level especially supporting the scale-up of FM training and awareness of the field. As a result of this work, in 2019, FM, for the first time, was included in the MCI syllabus for the undergraduate MBBS curriculum (Table 4, Quote 13). This is seen as a necessary step in propagating and thus institutionalizing FM to ensure exposure and awareness among undergraduate medical students deciding which career paths to pursue. There is still much work to be done, as it was only mandated that one hour is towards FM exposure.

In the next sections, we will explore contexts that served to either hinder or advance the motivations and implementation actions of innovators and early adopters.

### Factors that facilitate implementation

Greenhalgh Conceptual and Theoretical Bases for the Spread of Innovations help us understand the contextual factors that can facilitate the implementation process [45]. Facilitators were related to organizational contexts and readiness (e.g., academic institutions, who were willing to implement FM training programs), as well as related to outer contexts (e.g., the development of professional associations and inter-organizational networks) [45].

#### Organizational context and readiness for innovation [Greenhalgh]

To successfully implement a new field of training in an academic institution, participants reported a key to success was a receptive institutional context for change. The most significant number of FM programs in India are DNB programs implemented and run by private institutions. These same institutions were also the first to implement FM training. A common theme was that many of the first institutions to start FM were mission hospitals where FM’s principles aligned with the organization’s strategic vision (Table 5, Quotes 14 and 15). In summary, there was alignment between the aims of FM training, and the strategic vision and values of the organizations.

**Table 5:** Factors that facilitate implementation.

Quote Number	Quote	Participant
14	“CFH is a remarkable hospital in that it practices good quality care at an affordable cost to the poorer people, so that was the goal of the hospital and founder of the hospital”	Dr. Rajkumar Ramasamy, Christian Fellowship Hospital, Tamil Nadu
15	“The Christian Mission Hospital ideology or philosophy matches with FM…this hospital itself was built keeping in view the fishermen of this island’s surroundings… so the vision and the mission of this hospital was bound to treat them, so that is how they understood that FM could be an easier way to get into that philosophy.”	Dr. Resmi S. Kaimal, Lourdes Hospital, Kerala
16	“Unless and until you experiment, you are not going to gain anything. There are many people in this situation in India who say that there is no scope for FM and things like that. I have a supportive administration, supportive family, those two things have helped me to experiment with so many things, and I am interested in teaching, so for the sake of students, I do all these things so that they also get some exposure in these community clinics, house visits…camps also.”	Dr. Resmi S. Kaimal, Lourdes Hospital, Kerala
17	“There was not much awareness of FM at that time. Not among the public and not especially among the faculty in the college, because even though we are all medical persons, it was really surprising that many of the faculty did not know what FM was… So what I thought, was that the best thing would be to create awareness in every department… I went to almost all the departments which we need for FM like ENT, Ophthalmology, Pediatrics, Medicine, Surgery, … I went over there… I used to create an awareness about what is FM and what the PGs [postgraduates in FM] are supposed to learn from each of the departments. That was very helpful.”	Dr. M. Roshni, Calicut Medical College, Kerala
18	“This particular hospital is one place where the management has been recognizing our activities and understanding how valuable we are to the patients.”	Dr. Swapna Bhaskar, St. Philomena Hospital, Karnataka
19	“We also did our undergraduate training program because the vice-principal for UG [undergraduate] said FM should be there in the training program. It is not a requirement in the Medical Council of India…So, we developed this undergraduate training over two weeks, which was very much noticed by the institution because the student feedback was very good…People felt it was good and recently it has been extended into four weeks.”	Dr. Venkatesh S., Christian Medical College Vellore, Tamil Nadu
20	“Through conferences, mainly the AFPI conferences, national conference…so 2013 the first National [AFPI] Conference of FM was happening, and we all went to Delhi… I thought I will present about my department and I will be starting from scratch and just present what all we are doing. And after I presented someone came and told me that Dr. Sunil from CMC wants to see you … Then we discussed so many things, he was telling me this can be done, that can be done….”	Dr. Resmi S. Kaimal, Kerala
21	“PG [postgraduate] trainees also have been more aware of late because of WhatsApp and social media catching up. People in one part of the country are aware of the benefits available to PGs in another part of the country …PGs have been united. There are WhatsApp groups so they can discuss their issues and find out solutions.”	Dr. Bijayraj R., Kerala
22	“It was good to have a team, but I am also grateful to people from outside the country who were in touch with me who were keen about FM, so that really helped.”	Dr. Sunil Abraham, Tamil Nadu
23	“We still have a lot of international facilitators teaching, not that we cannot do that ourselves because we have done a lot of faculty development now… The reason why we have international family physicians still coming to our program is because we want our students to understand that this is actually a global specialty. It is not something that you have chosen because you did not get into medicine or surgery, or obstetrics. It is a thing which is so needed. You are doing something very important.”	Dr. Jachin Velavan, Tamil Nadu
24	“Half of the credit is due to the family physicians, but half of the credit is to the government also… Kerala’s primary care system or the government health system has been catering to people a good lot, not like in other states where the government is totally discarded, and it is the private hospitals who do most of the work, it is not like that in Kerala. Government doctors are also given their value, and government hospitals have their value in the public.”	Dr. Resmi S. Kaimal, Kerala
25	“The Government of Tamil Nadu had started seeing the big change that was happening in the [GP] private practitioners, so they asked, ‘Can this training be given?’. The attraction for them, was they don’t need to release their workforce from where they are, so that is the huge thing… so we started training the Tamil Nadu PHC doctors in the tribal areas. They are MBBS graduates. They have been working in the government system for many years, some of them like 15 years, ten years, and never had an update… So, the way we ran it was, we had ten mission hospitals, and there was one government hospital … They would be our hands-on training centres. So the candidates will do the modules at a distance, but they will come to these centres for hands-on training. It was a two-year course with 33 days of hands-on, so they would have to come 11 days at a time for three times. . . Then the Central Government, the NRHM [National Rural Health Mission], they asked, ‘Can you train our doctors?’ So they released funds for eight states … so we started training them.”	Dr. Jachin Velavan, Tamil Nadu

Additionally, it appears these institutions foster innovation by having a climate conducive to experimentation allowing pioneering family physicians to find ways to develop and implement new training programs (Table 5, Quotes 16 and 17). Experimentation ranged from trialling different ways of delivering care to patients, providing meaningful learning opportunities for students, and finding ways to educate other specialty programs and physicians about FM so they could better support FM education.

Organizational leaders played an important role to capitalize on the facilitators listed above. Strong institutional leadership with a vision in line with FM and supportive management to facilitate change were also essential facilitators that enabled pioneering family physician educators to break away from norms (Table 5, Quote 18). As described earlier, as of 2019, the MCI had mandated only one hour of FM training to undergraduate medical students. However, visionary institutions with supportive leadership, such as CMC Vellore had already implemented longer and more comprehensive FM rotations for their MBBS students’ years earlier (Table 5, Quote 19).

#### Outer context [Greenhalgh]

Interorganizational networks have been a critical facilitator for the spread of FM training and practice. Pioneering family physician educators spoke of the importance of forming teams, networking, and gaining support from others across their states and from other states. Networks supported sharing ideas and norms for implementing training programs, providing mentorship, and creating learning communities. Several participants spoke of the Academy of Family Physicians of India (AFPI), both nationally and state chapters, in facilitating networking among family physicians (Table 5, Quote 20). Multiple participants also shared how technology has expanded how practicing family physicians support one another, communicate with learners, and network in recent years (Table 5, Quote 21). Participants developing training programs also spoke of the benefit of international family physicians’ mentorship to share how FM functions in other contexts (Table 5, Quote 22). Similarly, participants shared those international collaborations helped trainees see FM as a valued global specialty (Table 5, Quote 23).

Although lack of government support is seen as a major barrier to FM implementation in India, discussed in the next section, this is not uniform nationally. Kerala and Tamil Nadu are described as States whose governments are primary care centred with receptive Ministries of Health (Table 5, Quote 24). This has helped facilitate the scale-up of training in their respective states and speaks to governments’ integral role in FM implementation success. For example, Kerala was the first state to approve an MD-FM program at Calicut Medical College in 2012, followed by Christian Medical College (CMC) Vellore in Tamil Nadu in 2017 –although CMC Vellore had first started a DNB-FM program in 1992. There also appears to be signs that other state governments are beginning to recognize the value of training and enhancing the skills of existing GPs in rural in remote communities (Table 5, Quote 25). In some cases, both states and the central governments have begun to fund the training of GPs to attend blended education programs in FM, like CMC’s PGDFM program.

### Factors that hinder implementation

Greenhalgh’s Conceptual and Theoretical Bases for the Spread of Innovations help us understand the factors that have hindered implementing FM in the Indian context to date. First, there are factors within organizations where there is a lack of “readiness to change” among peer groups [45]. However, the most significant obstacles seen relate to the outer context and policy where there is a perceived lack of government support for the implementation process [45].

#### Organizational context and readiness for innovation [Greenhalgh]

From Greenhalgh et al.’s model, successful implementation in an organizational context requires an assessment of implications to mitigate barriers and support advocacy [45]. With the introduction of FM came tension within institutions between specialist groups perceived to feel threatened by the emergence of FM and what this meant for collaboration (Table 6, Quotes 26 and 27). Although individual pioneering family physicians are trying to address this with organ-specific specialists within their respective institutions through peer education and discussion, there is still a broader lack of awareness of how FM is intended to integrate into the healthcare system and specialists’ work (Table 6, Quote 28).

**Table 6:** Factors that hinder implementation.

Quote Number	Quote	Participant
26	“People … did not want FM around. There was a lot of anti-FM lobby.”	Dr. Prince Christopher, Tamil Nadu
27	“Everybody was actually thinking, like, if you are learning ENT, that sort of encroaching feeling was there for them. But still repeatedly [I was] telling them and explaining to them as to what FM is. During my class, I was actually explaining about all those principles, and I was saying like this is actually sort of a three-tier system like all the primary they will be coming to us and we will be managing them but still if there is a need we will have to refer it to the concerned specialist. I was repeatedly explaining to them and making them aware that this is what we are doing. We are not concentrating on one specialty alone like we are not concentrating on General Medicine alone, we are not concentrating on OBG [obstetrics and gynecology] alone and convincing them that we are not concentrating on one specific specialty and somehow we were able to convince them.”	Dr. M. Roshni, Kerala
28	“A lot more advocacy is needed with the government and this tension between specialists and family physicians. That has to be resolved in a sensible way without signs of feeling threatened from either side or being that there is an unhealthy competition.”	Dr. Vijila Isac, Tamil Nadu
29	“One person who was a CMC alumnus…had an interview with one of his friends who was at the helm at the Medical Council of India, for a very short period of time, as an acting HOD… he was able to influence that man to think about FM. And that’s how FM got into the gazette of the list of specialty inside the MCI as a field of medical science. Without that happening, nothing would have happened.”	Dr. Prince Christopher, Tamil Nadu
30	“In 2002, the National Health Policy spoke about FM. They said there is a huge shortage of family physicians, 25% of postgraduate seats should be in FM, so that happened in 2002, but it was all pushed down. People didn’t want to hear about it. In 2010 the Government of India sent letters to all the medical schools to start MD FM.”	Dr. Sunil Abraham, Tamil Nadu
31	“At the national level, Dr. Raman Kumar has been putting in a lot of advocacy. He is putting the public interest litigation with the Supreme Court of India that the government is not implementing their health policies.”	Dr. Sunil Abraham, Tamil Nadu
32	“I think for it [FM] to succeed, the first thing that must be done is that the number of seats in FM has to be increased. You’ve got to have more family physicians trained in India. The way the government is allocating and giving recognition for the seats is ridiculous; in tertiary hospitals, in corporate hospitals… You recognize training in district hospitals and recognize family practices or primary health centers. The exam drives that learning. The exam is so robust that the people who study it have to learn what FM should require.”	Dr. Rajkumar Ramasamy, Tamil Nadu
33	“At the government level, there should be a lot of policy changes…there should be a department of FM in all the community health centres where training programs also happen for the FM graduates. Tertiary hospitals, there is hardly any requirement for FM in those kinds of hospitals, we should get out of tertiary hospitals, but missionary hospitals and medical colleges and all these are the places where FM needs to grow.”	Anonymous
34	“I am hoping that this training will be standardized across the country because currently there are some places which are good, some places are not good, so there has to be a standardization.”	Dr. Sunil Abraham, Tamil Nadu
35	“Standardization is another thing, standardization of premises, in the way of patient handling, treatment procedures. Right now, one is left to himself. There is no standardization worth talking about today. That is because the standards of MD education levels for medical graduates vary from person to person and from one medical college to the other.”	Dr. B.C. Rao, Karanataka
36	“Training institutions should insist that there should be an FM person in the department. Very few institutions have that. It is a mixture, like when I did it, it was a physician [internal medicine], surgeon, pediatrician, everything, everyone deciding what you are going to do, you have no role. I mean, a family physician need not be there to run an FM department? Which other department works like that? There should be a mandatory FM qualified person in a National Board accredited institution for FM and in more government medical colleges.”	Dr. Resmi S. Kaimal, Kerala
37	“They need to definitely have a proper training in the sense first thing they should understand what FM is, just by sending them to a place and get trained in FM is not what I mean by proper training…when I say proper training, I mean proper training by family physicians, not by specialists and super-specialists… still there is not enough training or FM aspirants by family physicians.”	Dr. Swapna Bhaskar, Karnataka
38	“In the community health centre, you have 30 beds, and that is where [in the current system] there is a requirement of having a surgeon, OB-GYN person, anesthetist, pediatrician, and an [internal medicine] physician. That is where we [AFPI Kerala] are saying that if you have a staff shortage like this, you can very well take a family physician there, where all these things can be done … that is what we are fighting for. Hopefully, it will work out in the long run, but the numbers are less…maybe in the long run, when these postgraduate courses [numbers] come up, it will work out.”	Dr. Resmi S. Kaimal, Kerala
39	“Right now, we had so many discussions with the health secretary as well as the health ministers, but now the problem is the number of qualified family physicians. It is very small.”	Dr. Serin Kuriakose, Kerala
40	“I could come back because I was already placed here. That is why I could come back. But, at the same time, the only thing, after post-graduation, family physicians are not getting that particular post of a family physician. But, at the same time, we can perform all skills what we have learned during three years of postgraduate course. And at the same time, we are getting the postgraduate allowance. That we are getting. Only thing we are lacking is that designation, specialist family physician.”	Dr. Serin Kuriakose, Kerala
41	“By the time I finished my graduation, even then, clarity was not there; where to join? Where I’ll fit in as a family physician? I liked the specialty. I liked the training. I enjoyed and was happy and very confident in the training also. But, where will I fit in? I didn’t know”.	Dr. Ally Plackal, Kerala
42	“Sometimes what happens is FM graduates, family physicians don’t understand that they have all these options, and they go and work as assistants in other departments. It is a situation pan India, everywhere people do that… one of my students who passed was moving to Maharashtra, Mumbai and she was searching for a job… I said, ‘What happened? You got some opening?’ She said, ‘No, madam, everyone is telling that you join as an assistant in urology, nephrology, neurology, then you can make your way from there.’ I said, ‘No way, three years you have passed, you have done your residency here, I don’t want to see you as an assistant in any other department’.”	Dr. Resmi S. Kaimal, Kerala
43	“No hospital in India had an understanding of what FM was. So, fitting back into a mainstream or into a hospital proved impossible. So, a few went back and did an Internal Medicine program just because they couldn’t find any FM openings and couldn’t push the concept of FM to potential employers.”	Dr. Malini Devanandan, Tamil Nadu
44	“For all this to succeed, we have to have a very defined career path for the youngsters. . . The government has to interfere, has to start a department, has to give them status, whether you like it or not young people want to be recognized in the community as well as in profession.”	Dr. B.C. Rao, Karanataka
45	“Make FM training compulsory for all those who want to practice it. It is ridiculous that a cardiologist or a general [internal medicine] physician cannot practice till they have done their MD [with postgraduate training], but you get people going to general practice without knowing what FM is. So all those entering it should be asked to do the degree, so you need to unequivocally have lots of people wanting to do FM because it is now a legal requirement.”	Dr. Rajkumar Ramasamy, Tamil Nadu

#### Outer context [Greenhalgh]

Participants perceived the most significant barrier as the lack of political will or government support for implementing FM in the Indian context. Central governments provided indications of support to promote FM through including FM as a recognized “speciality” under the MCI in 1984; and in the National Health Policy of 2002, indicating that 25 percent of all postgraduate training positions should be in FM or public health; and in 2010 recommending that state governments start MD-FM programs, but have had little impact as they did not include implementation plans. Stories from pioneers share that FM’s introduction on the MCI list may have resulted from a critical discussion instead of an organized central plan, supporting why there was little dissemination of this information (Table 6, Quote 29). FM advocates feel that the government should have mandated FM’s implementation instead of simply requesting it (Table 6, Quote 30).

### Recommendations to leverage implementation factors

It was apparent from participants that they felt an urgent need for government support to ensure FM growth (Table 6, Quote 31). Participants brought forward several recommendations related to educational policy reforms and health system-level reforms needed to ensure successful implementation.

FM training should be scaled up and implemented in a greater number of institutions with more training seats available (Table 6, Quote 32). Second, MCI regulations should be amended to support FM training needs, which is not a tertiary-based specialty (Table 6, Quotes 33). Currently, for new FM training programs to be accredited, they require a 30-bed inpatient unit in a tertiary center, aligning with subspecialty training requirements, not FM. Policy changes, such as those presented in the factors that facilitate implementation section for example, should allow for training in smaller community and district hospitals. Implementing these recommendations requires regulatory bodies to modify existing guidelines for program accreditation.

Additionally, a standardized system for FM education and accreditation for training programs is needed to ensure consistency and high-quality education for trainees across different FM programs, institutions, and states (Table 6, Quotes 34 and 35). One recommendation includes having a standardized national test that graduates complete offered by a regulatory board instead of individual institutions, ensuring standardization across institutions and degree types. Currently, the DNB-FM programs have a standardized national test. However, the MD-FM exams are specific to each university. Fourth, participants emphasize that family physicians should be leading FM training programs instead of other specialists, particularly as the number of family physicians increases (Table 6, Quotes 36 and 37). Finally, some participants spoke about the need to “grandfather” existing GPs into FM and the best way to do this. Currently, individual institutions offer distance or blended learning FM programs that target up-skilling existing GPs, such as the PGDFM program at CMC Vellore. However, presently the national regulatory boards (MCI and NBE) do not accredit those programs or individuals who complete these programs, which affords these graduates no FM status in the workplace post-graduation.

Healthcare delivery and funding are under state jurisdiction; early cohort physicians involved in developing state-level AFPI chapters have played an essential role in advocating for service delivery changes. AFPI Kerala has been particularly successful in working with its receptive state government to advocate for family physicians’ positions in the government-run primary care sector. Currently, government-run community health centres (CHCs) are meant to be staffed by four medical specialists; however, finding specialists to work in these centres is a great challenge, resulting in many being closed. AFPI Kerala has advocated that a single-family physician may be able to provide the services of these four specialists (Table 6, Quote 38). The current bottleneck in implementing this change is having enough trained family physicians to take on these positions (Table 6, Quote 39). Presently, there are few examples of graduates working in government CHCs, typically because they worked in these centres before their postgraduate FM training as GPs (Table 6, Quote 40). However, they do so without the designation or status of a specialist in FM when they return.

The lack of job opportunities and family physician specialty status is not simply seen in the government sector but more broadly across the healthcare system. Several participants spoke about the lack of positions for family physicians post-graduation (Table 6, Quote 41). Training produces highly skilled family physicians with a broad scope, but they have difficulty finding jobs that match their qualifications. This results in lost resources through a mismatch in skills and opportunities (Table 6, Quote 42). Individuals sometimes choose to leave the discipline altogether (Table 6, Quote 43). Some participants shared that they were left forging and creating positions in different contexts; one participant is the family physician for a school population, including all the students, employees, and often their families. Another participant found a position within a hospital specializing in mental health, where she works collaboratively with psychiatrists.

The same continues to be a problem today. FM leaders are looking to the governments to help support larger-scale policy changes to help support greater dissemination of the field by raising awareness of FM and creating job opportunities in line with skill sets (Table 6, Quote 44). Not creating career opportunities for graduates and ensuring government professional recognition risks impeding FM growth and attracting students. Some participants believe that policy changes are needed to mandate that postgraduate FM training is required to practice as a physician in primary care, no different from any other specialty as a means to support growth of the field (Table 6, Quote 45).

## Discussion

To our knowledge, this is the first study exploring the implementation of FM in India. Using Rogers’ Diffusion of Innovation Theory and Greenhalgh’s Conceptual and Theoretical Bases for the Spread of Innovations and Model of Diffusion, we highlight how FM has emerged [45, 48].

### Innovators and early adopters

We focus on the role of innovators and early adopters in the implementation process. We see the work of multiple independent innovators—individuals willing to take risks to develop new ideas and try new forms of practice [48]—who were not necessarily family physicians. They recognized the need for change in the healthcare system. They believed a shift in medical education towards community-focused primary care was needed. Many innovators described realizing that FM embodied what they hoped to achieve from their experiences or exposures to countries with family physicians integrated into their health systems. Through the work of innovators, various types of postgraduate training programs were developed and implemented. First, the DNB in FM full-time residency training programs in private institutions. Then, part-time, blended training programs with both distance and in-person training. Finally, the MD in FM, a full-time residency training program, was implemented in medical colleges in India.

Individuals willing to enroll and study in these new FM training programs are considered early adopters. Early adopters often take on leadership roles, embrace change opportunities and are aware of the need to change, so they are comfortable adopting new ideas [48]. Early adopters in our study highlight how they also act as innovators to help spread or implement FM in new settings and educational institutions. This concept of early adopters, “re-inventing” innovation, is not new and was recognized by Rogers as a critical component of disseminating and adopting innovations [69, 70]. Reinvention is the degree to which the adopter changes an innovation in the adoption process. In implementation science, and as highlighted in Greenhalgh’s Model of Diffusion, many believe that for innovation adoption, individuals need to have sufficient information about the innovation, how it is used and how it affects them personally before they are willing to adopt it [45, 71]. This was not necessarily true for early adopters in our study and perhaps speaks to the "tension for change" that existed [72].

Our research is unique in that it specifically explored the role of early cohort family physicians in the FM implementation process. We specifically sought to understand how these pioneers created an environment capable of supporting FM. Our study found that early cohort family physicians have supported implementing FM through their roles as leaders and champions, educators and teachers, and advocates. We find similar themes when we extrapolate our findings to literature in other LMIC. In Brazil, family physician faculty were similarly seen as crucial for demonstrating their role in the healthcare system and acting as role models who can “teach with resolve and competence [to] awaken a sense of vocation in their students” [73]. Research from multiple other LMIC countries highlights the integral role that FM champions play in implementation [19, 74].

### Factors that facilitate and hinder implementation

We found academic institutions and professional organizations were important facilitators in the FM implementation process in India. Institutions provided a natural environment that promoted experimentation and facilitated the development of leaders, advocates, and teachers. Many of the first institutions to successfully offer FM training programs over time in India were private, faith-based hospitals where the institutions underlying vision and mission aligned with the principles of FM. These institutions often had a desire to provide care to underserved populations. In India itself, many hospitals and universities have started and subsequently stopped FM training programs, suggesting much variability in environments. In other contexts, such as Uruguay, we see that early cohort family physicians, FM champions, or leaders have spent many years or decades overcoming institutional resistance. In Uruguay, Universities administer professional degrees, and the University of Republic provides 90% of medical training in the country. Although the FM residency program was accepted and formalized by the National Technical Commission, it was not accepted by the University of Republic, so the institution’s first cohorts of FM graduates were denied existence [24]. This resistance may have existed in some institutions in India where FM programs could not sustain themselves. Greenhalgh’s Model supports this finding highlighting how institutions with solid leadership with a clear strategic vision provide environments conducive to experimentation and innovation adoption [45].

The development of professional organizations such as the AFPI has facilitated the spread of FM by providing a space for innovators and early adopters to come together, network, learn, share ideas and norms, and refine their programs through natural peer mentorship. This supports Greenhalgh’s model that highlights the importance of the outer context in supporting the spread of health innovations [45]. Interestingly, Greenhalgh’s model suggests that interorganizational models generally support adoption once an innovation is perceived as “the norm,” which was not the case in India [45]. In this research, participants describe the AFPI as a significant facilitator of FM implementation. AFPI has brought together FM leaders and champions’ voices, increasing the call for action from central and state governments. They ask for support to scale-up FM training, standardize and recognize training, and health system changes needed to integrate FM. Professional organizations, such as AFPI, were vital for advocacy and creating a space for family physicians to network and learn. Professional organizations’ important role in implementing FM is seen in multiple countries globally, including Canada, Sri Lanka, and Uganda, to name a few [14, 75, 76]. Congruent with our research, it has been found that FM specific professional organizations support advocating that FM should be an independent discipline, the development of training and continuing medical education [77].

One important finding in our research was the significant role the AFPI played in networking, and creating a space where early cohort family physicians felt they “belonged.” Social relationships are reflected in many implementation theories, including Rogers’ theory, where individuals and how they interact with one another (innovators and early adopters) are at the core of influencing and supporting information exchange and dissemination [48]. From the social psychological analysis of group processes, it is central that people derive part of their self-concept from the social groups and categories they belong to [78, 79]. They define themselves through their social identity, which is “an individual’s knowledge that he belongs to certain social groups together with the some emotional and value significance to him or his group membership” [78, 79]. Similarly, transformational leaders within these organizations or social groups can transform individual action into group action, as seen in the India example as well [79]. Less articulated in the FM implementation literature is the power of professional organizations in supporting first or early cohort family physicians, or early adopters, in developing their sense of “self-identity.” However, we believe this is a common sentiment as the literature frequently identifies a “lack of awareness of the field” as a barrier to FM implementation [80, 81]. Our research, and the role of the AFPI in bringing together early cohort family physicians, suggest that implementing professional organizations early in the implementation process may help support early cohort family physicians during this period in developing a sense of identity.

Similarly, peer networks were found to be particularly important given that early cohort family physicians often faced much resistance to change, particularly from other medical specialists, who lacked awareness of the field of FM or felt threatened by its implementation and its implications on their own specialty fields. The resistance by medical specialties is not unique to India and is seen as a barrier to FM implementation in two reviews; the first includes Brazil, Haiti, Indonesia, Kenya and Mali [19]; and the second includes Ghana, Nigeria, the Democratic Republic of Congo, Rwanda, Uganda, Kenya and Tanzania [81]. Greenhalgh’s Model speaks to how assessing implications within organizations or institutions is essential for innovation adoption and addressing them for successful implementation. In our examples, some early cohort graduates spoke of how they handled this at the institutional level. They provided education sessions to various specialists’ programs to educate them on what FM was, how they could work collaboratively, and how FM could benefit their work. Thus, they mitigated fears that FM may affect specialists’ practice or patient population, which proved to be valuable. This finding is congruent with literature emphasizing that successful health system change requires system stakeholders and clarifying roles to avoid conflict and concerns about overlapping scope [82, 83]. Barriers to FM implementation, including lack of understanding of the field from stakeholder groups and stakeholder buy-in, go hand in hand. Commitment to goals from different groups is relative. Shared purpose and understanding among stakeholder groups are important to successful implementation [84].

### From diffusion to dissemination

Using Greenhalgh’s model, we see that implementation of FM in India from the early 1980s to the present has predominantly occurred through passive diffusion or a “let it happen” approach, which has been an emergent process that is adaptive and primarily driven by individual leaders, their actions and their social networks as opposed to policy [45, 85]. Due to the emergent process of FM implementation in India, FM training to date has arisen in multiple ways with several different types of training programs across various institutions, including DNB-FM, MD-FM, PGDFM and others. Participants share how the implementation of these training programs has been driven by individuals, specifically innovators and early adopters, looking to address gaps in the healthcare system. Greenhalgh’s model supports what was seen in India, where the organizational context and specifically receptive institutions provided the nurturing environment for innovation instead of government-sanctioned implementation. This decentralized approach has led to many variations across programs and offered innovators opportunities to experiment with program design.

### Government role in supporting implementation

Although this approach has made significant progress since the early 1980s, there is an imperative need for government support at this time. To continue towards the path of institutionalization, active and directed support—“make it happen” or “help it happen” approaches—are needed by policymakers and governments to formally disseminate FM further and create pathways for greater integration of family physicians into the existing healthcare system. This includes: (1) increasing the number of FM postgraduate training seats in India which promotes having skilled providers in the primary care sector; (2) increasing the mandated amount of FM training in the undergraduate MBBS curriculum to match those of other core specialty subjects (such as pediatrics, internal medicine, and general surgery, for example) to ensure students are aware of the specialty; (3) ensure job opportunities available for family physicians in the health sector match their skills and the needs of the community, specifically, State governments should consider the benefits of creating specialist family physician positions in CHCs. The 2022 Indian Public Health Standards recommends that one of the specialists in a CHC be an FM specialist [86]; however, it is ultimately at the discretion of state governments to implement this recommendation.

India produces approximately 65,000 undergraduate medical graduates annually but only offers 10,000 specialty postgraduate seats [87]. That leaves 85% of medical school graduates without postgraduate training in any field. Governments and policymakers may wish to consider increasing the number of FM postgraduate training seats in India to promote having skilled providers in the primary care sector. In 2023, only 110 DNB -FM training spots were available nationally [88]. This number has fluctuated over the last few decades as new programs begin and old programs cease. In addition, only five government medical colleges, of a total of 286, offer the MD-FM program as of 2021 [89]. These numbers suggest that just over 1% of all postgraduate seats in India are in FM. Examples of countries with family physicians as the first point of access in their healthcare systems include Canada, Australia, and New Zealand. We looked at these three countries to assess the proportion of family physicians versus other specialists and the annual number of postgraduate training seats in FM. In Canada, as of 2019, there were 241 physicians per 100,000, of which 122 (51%) were family physicians, and 119 (49%) were specialists [90]. In Australia and New Zealand, the term GP describes physicians who have completed postgraduate training in primary care. In Australia, as of 2019, 3594 (45%) out of 7893 postgraduate training seats were for postgraduate training in primary care (general practice) [91]. In New Zealand, as of 2018, specialists make up 39% of all physicians, while GPs make up 28%, and trainees make up the remainder [92]. As of 2019, 3,671 (38%) of 9683 postgraduate training seats in New Zealand were for general practice [92].

The need for government support is not unique to India. It is an essential component of FM implementation in a country context. Research from an international survey of FM training programs highlights the critical role governments play in FM implementation through financing medical education, determining the number of specialty training positions, and, relatedly the number and location of programs [20]. Similarly, a scoping review of FM implementation in Sub-Saharan Africa highlights the need for government support to address the slow pace at which family physicians are trained and integrated into the healthcare system [93].

The homegrown nature of FM in India appears to be unique. This research highlights how individuals and institutions within India have supported the spread of FM training through faculty development and implementation support within their own country. In the literature from other LMICs, much of the support is from international peers working directly with institutions or governments to support the initial implementation of FM [19, 81]. This divergence highlights the multiple ways FM can originate in a country. The initial instigator of FM implementation may be from local innovators and pioneering individuals (bottom-up) as seen in India and Venezuela [20], governments (top-down) as seen in countries like Vietnam [94], Turkey [95] and Kuwait [13], or international peer collaborators working with institutions and governments seen in Ethiopia [96], Uganda [18] and Nepal [97]. Although international collaborators may not have initiated FM in India, our study’s findings acknowledge their support. International collaborators support FM’s legitimacy as a global specialty and provide important mentorship to Indian pioneering family physicians through one-on-one support.

What can be said about the Indian context is that the last four decades have allowed pioneering family physicians to develop, implement and establish training programs and highlight a desire for FM among the medical community. Decades of experience with existing training programs enables governments to more easily support training implementation and scale-up when several successful programs are in place to model from, strong leaders in the field to help facilitate further spread, and a base of graduates who can teach and mentor future generations.

Globally, to drive FM implementation forward, we need a stronger evidence base to understand the role of FM in strengthening primary care and PHC. There is currently an evidence gap in this area [98]. The number of countries choosing to implement FM is perhaps a subtle sign of its utility [98]. This research highlights that one critical period to study is when countries first implement FM, particularly the first decades when the initial cohorts of family physicians are trained and placed in a system that does not recognize their role, skills, or contributions. Time is needed for these early family physicians to build an administrative structure in FM, including training others; work with policymakers to identify where in the healthcare system they are best suited; work with other community-level health care providers, and build relationships with their communities and patients. This research contributes to understanding the potential value of FM as one model of delivering primary care. This paper explored how FM is implemented in a national context. Our second paper will propose potential mechanisms by which family physicians strengthen PHC.

### Strengths and limitations

This study is based on twenty in-depth interviews, eighteen of which were done in person, in the settings where participants live and work, across three states. Our sample represented family physicians working in urban, rural and academic settings and explored the implementation of FM in three unique States. This diverse representation supports transferability of findings to similar settings and regions. The data includes recorded interview transcripts, field notes, and reflections made after each interview, incorporated into the data analysis. This study used several techniques to promote rigour and credibility [62]. Rigour was ensured using theoretical constructs for study design and analysis, researcher immersion in the field, and memoing and producing field notes. Credibility was sought using codebook validation with a peer researcher and member checking. Member reflections were also integrated into the codebook. Multivocality was achieved by ensuring participatory research methods and having participants as research collaborators.

This study is also subject to several limitations. Given the lack of documentation of FM training programs, authors cannot say with certainty which institution first implemented FM training in India despite best efforts to understand this from pioneers and accrediting institutions. With confidence, we can say that the implementation of FM has been most significant in these three states since the early 1990s, and Christian Fellowship Hospital, along with CMC Vellore, were the first institutions in Southern India to implement postgraduate training in FM. Given the nature of the questions, responses may be subject to recall bias. Finally, in this study, we interviewed those who achieved FM accreditation through full-time postgraduate training (DNB) and the alternative route before implementing postgraduate training (MNAMS). However, we did not interview any blended part-time distance-based learning program graduates. Including graduates from this third group of trainees may have offered additional insights into varying motivations and skills gained from an alternative form of training and different facilitators and barriers to integration within the existing healthcare system. India is a diverse country. Although FM emerged in Southern India, we expect each state to have its own pioneers and emergence stories as FM continues to spread nationally. Finally, many recommendations for FM implementation revolve around the need for greater government involvement. As such, future research could strengthen these findings by including views from policymakers and government stakeholders.

## Conclusions

The implementation of FM in a country is complex and lengthy. Over the first four decades in India, implementation has occurred through passive diffusion mediated through innovators and pioneering family physicians who are seen as champions in implementing training programs. The field’s continued growth occurred through peer-mediated spread, mentorship, supportive academic institutions and the development of professional organizations. India is now at a stage where formal dissemination programs supported by governments and policymakers are needed. To ensure that FM can have the intended impact on strengthening PHC, pioneering family physicians agree that governments should support scaling up FM training both at undergraduate and postgraduate levels, support policies that ensure training match the needs of the primary care system and create pathways for family physicians to be integrated into the existing healthcare system.

## References

[1] World Health Organization and United Nations Children’s Fund.Declaration of Astana. Global Conference on Primary Health Care; 2018; Astana, Kazakhstan: World Health Organization and United Nations Children’s Fund (UNICEF).

[2] World Health Organization and United Nations Children’s Fund. A vision for primary health care in the 21st century: towards universal health coverage and the Sustainable Development Goals. Geneva: World Health Organization and the United Nations Children’s Fund (UNICEF); 2018 (WHO/HIS/SDS/2018.15). Contract No: Licence: CC BY-NC-SA 3.0 IGO.

[3] Gupta A, Steele Gray C, Landes M, Sridharan S, Bhattacharyya O. Family Medicine–An Evolving Field Around the World. Canadian Family Physician. 2021:647–51. doi: 10.46747/cfp.6709647 34521704 PMC9683363

[4] Starfield B. Is primary care essential? The Lancet. 1994;344(8930):1129–33. doi: 10.1016/s0140-6736(94)90634-3 7934497

[5] Starfield B, Shi L, Macinko J. Contribution of primary care to health systems and health. Milbank Quarterly. 2005;83(3):457–502. doi: 10.1111/j.1468-0009.2005.00409.x 16202000 PMC2690145

[6] Kidd M. The contribution of family medicine to improving health systems: A guidebook from the world organization of family doctors. Second ed. London: Radcliffe Publishing; 2013.

[7] Arya N, Gibson C, Ponka D, Haq C, Hansel S, Dahlman B, et al. Family medicine around the world: overview by region. Canadian Family Physician. 2017;63(6):436. 28615392 PMC5471080

[8] Mash R, Reid S. Statement of consensus on Family Medicine in Africa. African Journal of Primary Health Care and Family Medicine. 2010;2(1).

[9] Bentzen BG, Bridges-Webb C, Carmichael L, Ceitlin J, Feinbloom R, Metcalf D, et al. The Role of the General Practitioner/Family Physician in Health Care Systems: A Statement from WONCA. 1991.

[10] Canadian Institute for Health Information. Continuity of Care With Family Medicine Physicians: Why It Matters. Ottawa, Ontario: CIHI; 2015.

[11] Glazier RH, Moineddin R, Agha MM, Zagorski B, Hall R, Manuel DG, et al. The impact of not having a primary care physician among people with chronic conditions. Toronto: Institute for Clinical Evaluative Studies; 2008.

[12] Strumpf E, Ammi M, Diop M, Fiset-Laniel J, Tousignant P. The impact of team-based primary care on health care services utilization and costs: Quebec’s family medicine groups. Journal of Health Economics. 2017;55:76–94. doi: 10.1016/j.jhealeco.2017.06.009 28728807

[13] Abyad A. Family Medicine in the Middle East: Reflections on the Experiences of Several Countries. The Journal of the American Board of Family Practice. 1996;9(4):289–97. 8829080

[14] Ramanayake RPJC. Historical evolution and present status of family medicine in Sri Lanka. Journal of Family Medicine and Primary Care. 2013;2(2):131–4. doi: 10.4103/2249-4863.117401 24479065 PMC3894032

[15] Ackerman L, Karki P. Family practice training in Nepal. Family Medicine. 2000;32(2):126–8. 10697772

[16] Prueksaritanond S, Tuchinda P. General Practice Residency Training Program in Thailand: Past, Present, and Future. J Med Assoc Thai. 2001;84:1153–7. 11758852

[17] Udonwa NE, Ariba A, Yohanna S, Akin-Moses L. Family Medicine in West Africa: progress, milestones, and challenges so far in Nigeria (1980–2010). Nigerian Journal of Family Practice. 2011;1(2).

[18] Ssenyonga R, Seremba E. Family medicine’s role in health care systems in sub-saharan Africa: Uganda as an example. Family Medicine. 2007;39(9):623–6. 17932794

[19] Rouleau K, Bourget M, Chege P, Couturier F, Godoy-Ruiz P, Grand’Maison PH, et al. Strengthening Primary Care Through Family Medicine Around the World Collaborating Toward Promising Practices. Family Medicine. 2018;50(6):426–36. doi: 10.22454/FamMed.2018.210965 29537479

[20] Haq C, Ventres W, Hunt V, Mull D, Thompson R, Rivo M, et al. Where there is No Family Doctor: The Development of Family Practice around the World. Academic Medcine. 1995;70(5):370–80. doi: 10.1097/00001888-199505000-00012 7748381

[21] Roberts R. From the President: The theme is team—Primary Health Care in Chile: WONCA; 2011 [cited 2021 August 30th]. Available from: https://www.globalfamilydoctor.com/news/fromthepresidentthethemeisteam-primaryhealthcareinchile.aspx.

[22] Montegut A, Cartwright CA, Schirmer JM, Cummings S. An International Consultation: The Development of Family Medicine in Vietnam. Family Medicine. 2004;36(5). 15129383

[23] National Board of Examinations. Guidelines for Competency Based Training Programme in DNB-Family Medicine. Medical Enclave, Ansari Nagar, New Delhi: 2021.

[24] Ponzo J. Family and Community Medicine in Uruguay from 1997 to 2019: how many kilometers will it take to reach that town? Cien Saude Colet 2020;25(4):1205–14. 32267423 10.1590/1413-81232020254.29332019

[25] Mash R, Howe A, Olayemi O, Makwero M, Ray S, Zerihun M, et al. Reflections on family medicine and primary healthcare in sub-Saharan Africa. BMJ Global Health. 2018;3(Suppl 3). doi: 10.1136/bmjgh-2017-000662 29765778 PMC5950631

[26] Bhore Committee. Report of the Health Survey and Development Committee. 1946. Available from: https://www.nhp.gov.in/bhore-committee-1946_pg.

[27] Rao M, Rao KD, Shiva KumarAK, Chatterjee M, Sundararaman T. Human resources for health in India. Lancet. 2011;377:587–98. doi: 10.1016/S0140-6736(10)61888-0 21227499

[28] Das J, Daniels B, Ashok M, Shim E-Y, Muralidharan K. Two Indias: The structure of primary health care markets in rural Indian villages with implications for policy. Social Science & Medicine. 2020;(112799). doi: 10.1016/j.socscimed.2020.112799 32553441 PMC9188269

[29] Mackintosh M, Channon A, Karan A.,Selvaraj S., Cavagnero E., Zhao H. What is the private sector? Understanding private provision in the health systems of low-income and middle-income countries. The Lancet 2016;388:596–605. 10.1016/S0140-6736(16)00342-1 27358253

[30] Karan A, Selvaraj S, Mahal A. Moving to universal coverage? Trends in the burden of out-of-pocket payments for health care across social groups in India, 1999–2000 to 2011–12. PLoS One. 2014;9:e105162. doi: 10.1371/journal.pone.0105162 25127454 PMC4134256

[31] Ravi S, Gupta D, Williams J. Reconstructing the Medical Council of India. SSRN. 2017. doi: 3041204

[32] Government of India. National Health Profile of India—2009: Central Bureau of Health Intelligence; 2009.

[33] Das J, Holla A, Das V, Mohanan M, Tabak D, Chan B. In Urban And Rural India, A Standardized Patient Study Showed Low Levels Of Provider Training And Huge Quality Gaps. Health Affairs. 2011;31(12):2774–84. 10.1377/hlthaff.2011.1356 PMC3730274 23213162

[34] Government of India. National Health Policy 2017. New Delhi: Ministry of Health and Family Welfare; 2017.

[35] Government of India. Guidelines for Health and Wellness Centres in India. New Delhi: Ministry of Health and Family Welfare; 2019.

[36] Ramani S, Sivakami M, Gilson L. How context affects implementation of the Primary Health Care approach: an analysis of what happened to primary health centres in India BMJ Global Health. 2019;3. 10.1136/bmjgh-2018-001381 PMC6626469 31354968

[37] Tikkanen R, Osborn R, Mossialos E A. D, Wharton GA. International Health Care System Profiles: India. The Commonwealth Fund; 2020.

[38] Government of India. Indian Public Health Standards (IPHS) for Primary Health Centres Guidelines. Dictorate General of Health Services, Ministry of Health and Family Welfare; 2006.

[39] Sharma DC. India still struggles with rural doctor shortages. The Lancet. 2015;386(10011):2381–2. doi: 10.1016/S0140-6736(15)01231-3 26700521

[40] Government of India. Comprehensive Primary Health Care: National Health Systems Resource Centre, Ministry of Health and Family Welfare, Government of India,; 2021 [cited 2021 August 28]. Available from: https://nhsrcindia.org/comprehensive-primary-health-care.

[41] Lahariya C. Health & Wellness Centers to Strengthen Primary Health Care in India: Concept, Progress and Ways Forward. Indian J Pediatr. 2020;87(11):916–29. 32638338 10.1007/s12098-020-03359-z PMC7340764

[42] Government of India. Ayushman Bharat: Comprehensive Primary Health Care through Health and Wellness Centers Operational Guidelines. Department of Health and Family Welfare; 2018.

[43] Nilsen P. Making sense of implementation theories, models and frameworks. Implementation Science. 2015;10(53). 25895742 10.1186/s13012-015-0242-0 PMC4406164

[44] Rabin BA, Brownson RC. Developing the Terminology for Dissemination and Implementation Research. In: Brownson R.C.; Colditz GAP, E.K., editor. Dissemination and Implementation Research in Health. New York: Oxford University Press; 2012. p. 23–51.

[45] Greenhalgh T, Robert G, Macfarlane F, Bate P, Kyriakidou O. Diffusion of innovations in service organizations: systematic review and recommendations. Milbank Quarterly. 2004;82(4):581–629. doi: 10.1111/j.0887-378X.2004.00325.x 15595944 PMC2690184

[46] Freidson E. Professional Powers—A Study of the Institutionalization of Formal Knowledge. United States: The University of Chicago Press; 1988.

[47] Lancet The. The Astana Decleration: the future of primary health care? The Lancet. 2018;392. 10.1016/S0140-6736(18)32478-4. 30343840

[48] Rogers EM. Diffusions of Innovations. 5th Ed. New York: Free Press; 2003.

[49] Grinyer A. The Anonymity of Research Participants: Assumptions, Ethics and Practicalities. Social Research Update. 2002(36).

[50] Stark JL. The Potential of Deweyan-Inspired Action Research. Education and Culture. 2014;30(2):87–101.

[51] Greenwood DJ. Pragmatic Action Reserach. International Journal of Action Research,. 2007;3(1+2):131–48.

[52] Bradshaw C, Atkinson S, Doody O. Employing a Qualitative Description Approach in Health Care Research. Global Qualitative Nursing Research. 2017;4:1–8. doi: 10.1177/2333393617742282 29204457 PMC5703087

[53] Caelli K, Ray L, Mill J. “Clear as mud”: Toward greater clarity in generic qualitative research. International Journal of Qualitative Methods. 2003;2(2):1–23.

[54] Sullivan-Bolyai S, Bova C, Harper D. Developing and refining interventions in persons with health disparities: The use of qualitative description. Nursing Outlook. 2005;53:127–33. doi: 10.1016/j.outlook.2005.03.005 15988449

[55] Neergaard MA, Oleson F, Anderson R, Sondergaard J. Qualitative description—The poor cousin of health research? BMC Medical Research Methodology. 2009;9(52). doi: 10.1186/1471-2288-9-52 19607668 PMC2717117

[56] Schultz PR, Meleis AI. Nursing epistemology: Traditions, insights, questions. Journal of Nursing Scholarship. 1988;10:217–21. doi: 10.1111/j.1547-5069.1988.tb00080.x 3203945

[57] Sandelowski M. Whatever Happened to Qualitative Description? Research in Nursing & Health. 2000;23:334–40. doi: 10.1002/1098-240x(200008)23:4&lt;334::aid-nur9&gt;3.0.co;2-g 10940958

[58] Clancy M. Is reflexivity the key to minimising problems of interpretation in phenomenological research? Nurse Researcher. 2013;20(6). doi: 10.7748/nr2013.07.20.6.12.e1209 23909106

[59] Patton MQ. Qualitative Evaluation and Research Methods. Beverly Hills, CA: Sage; 1990.

[60] Parse RR. Qualitative inquiry: The path of sciencing. Sudbury, MA: Jones & Bartlett; 2001.

[61] Fawcett JG J. Evaluating research for evidence based nursing practice. Philadelphia: F.A. Davis; 2009.

[62] Tracy SJ. Qualitative Quality: Eight "Big Tent" Criteria for Excellent Qualitative Research. Qualitative Inquiry. 2010;16(10):837–51.

[63] Ironside PM. Using narrative pedagogy: Learning and practising interpretive thinking. Issues and Innovations in Nursing Education,. 2006;55:478–86. doi: 10.1111/j.1365-2648.2006.03938.x 16866843

[64] DeJonckheere M, Vaughn LM. Semistructured interviewing in primary care research: a balance of relationship and rigour. Family Medicine and Community Health. 2019;7(2). doi: 10.1136/fmch-2018-000057 32148704 PMC6910737

[65] Phillppi J, Lauderdale J. A Guide to Field Notes for Qualitative Research: Context and Conversation. Qualitative Health Research. 2018;28(3):381–8. doi: 10.1177/1049732317697102 29298584

[66] Hunt M. Strengths and Challenges in the Use of Interpretive Description: Reflections Arising From a Study of the Moral Experience of Health Professionals in Humanitarian Work. Qualitative Health Research. 2009;19(9):1284–92. doi: 10.1177/1049732309344612 19690208

[67] Strauss AL. Qualitative analysis for social scientists. New York: Cambridge University Press; 1987.

[68] Baxter P, Jack S. Qualitative Case Study Methodology: Study Design and Implementation for Novice Researchers. The Qualitative Report. 2008;13(4):544–59.

[69] Rogers EM, editor Re-invention during the innovation process. Presented at the Workshop on Assessment of Current Developments on the Diffusion of Innovations, Northwestern University, November 15–16 (revised); 1978.

[70] Rice R, Rogers EM. Reinvention in the Innovation Process. Knowledge. 1980;1(4):499–514.

[71] Hall GE, Hord SM. Change in Schools. Albany: Sate University: New York Press; 1987.

[72] Gustafson DH, Sainfort F, Eichler M, Adams L, Bisognano M, Steudel H. Developing and Testing a Model to Predict Outcomes of Organizational Change. Health Services Research. 2003;38(2). 10.1111/1475-6773.00143 PMC1360903 12785571

[73] Blasco P, Levites M, Janaudis M, Moreto G, Roncoletta A, de Benedetto M, et al. Family medicine education in Brazil: challenges, opportunities, and innovations. Academic Medicine. 2008;2008(7):684–90. doi: 10.1097/ACM.0b013e3181782a67 18580090

[74] Mash RJ, de Villiers MR, Moodley K, Nachega JB. Guiding the Development of Family Medicine Training in Africa Through Collaboration With the Medical Education Partnership Initiative. 2014;89(8):S73–S7. doi: 10.1097/ACM.0000000000000328 25072584

[75] College of Family Physicians of Canada. A Brief History of the College [cited 2019 July 18th]. Available from: https://familymedicineheritage.ca/college-history/.

[76] Namatovu J, Haq C. Development of family medicine in Uganda: WONCA Global Family Doctor; 2016 [cited 2019 July 23rd]. Available from: https://www.globalfamilydoctor.com/News/DevelopmentofFamilyMedicineinUganda.aspx.

[77] Kidd M. WONCA and the new UN Sustainable Development Goals 2015. Available from: https://www.wonca.net/News/FromthePresidentWONCAandthenewUnitedNationsSustainableDevel.aspx.

[78] Tajfel H. Social categorization. English manuscript of ‘‘La cate´gorisation sociale.”. In: Moscovici S, editor. Introduction a`la psychologie sociale. 1. Paris: Larousse; 1972. p. 272–302.

[79] Hogg MA, Reid SA. Social Identity, Self-Categorization, and the Communication of Group Norms. Communication Theory. 2006;16(1):7–30.

[80] Moosa S, Downing R, Mash B, Pentz S, Essuman A. Understanding of family medicine in Africa: a qualitative study of leaders’ views. British Journal of Medical Practice. 2013:e209–e16. doi: 10.3399/bjgp13X664261 23561788 PMC3582980

[81] Flinkenflogel M, Essusman A, Chege P, Ayankogbe O, De Maeseneer J. Family medicine training in sub-Saharan Africa: South-south cooperation in Primafamed project as strategy for development. Family Practice. 2014;31(4). 10.1093/fampra/cmu014 PMC4106404 24857843

[82] Best A, Greenhalgh T, Lewis S, Saul JE, Carroll S, J B. Large-system transformation in health care: a realist review. Milbank Quarterly. 2012;90(3):421–56. doi: 10.1111/j.1468-0009.2012.00670.x 22985277 PMC3479379

[83] World Health Organization. Leading health system transformation to the next level. Durham, United Kingdom: World Health Organization, Regional Office for Europe; 2017.

[84] Eveland JD. Diffusion, Technology Transfer, and Implementation: Thinking and Talking about Change. Knowledge. 1986;8(2):303–22.

[85] Fonesca J. Complexity and Innovation in Organizations. London: Routledge; 2001.

[86] Ministry of Health and Family Welfare Government of India. Indian Public Health Standards: Community Health Centre. New Delhi, India: National Health Mission; 2022.

[87] Kumar R. Frequently asked questions about family medicine in India. Journal of Family Medicine and Primary Care. 2016;5(1):3. doi: 10.4103/2249-4863.184615 27453835 PMC4943145

[88] National Board of Examinations. NBE Accredited Seats: Family Medicine: All States 2023 [updated Jan 10, 2023; cited 2023 Jan 10]. Available from: https://accr.natboard.edu.in/online_user/frontpage.php?v=4.

[89] Mishra MK. Total number of Medical Colleges and MBBS Seats in India, know the details: Shiksha; 2021 [cited 2021 July 7th]. Available from: https://www.shiksha.com/medicine-health-sciences/medicine/articles/total-number-of-medical-colleges-and-mbbs-seats-in-india-know-the-details-blogId-54817.

[90] Canadian Institute for Health Information. Physicians in Canada, 2019 Ottawa, ON: CIHI; 2020.Available from: https://www.cihi.ca/sites/default/files/document/physicians-in-canada-report-en.pdf.

[91] Australian Government Department of Health. Medical Education and Training (MET): Australian Government Department of Health; 2021. Available from: https://hwd.health.gov.au/resources/publications/report-met4-2019.pdf.

[92] Medical Council of New Zealand. The New Zealnd Medical Workforce in 2018. 2019.Available from: https://www.nzdoctor.co.nz/sites/default/files/2019-12/Workforce-Survey-Report-2018.pdf.

[93] Flinkenflögel M, Sethlare V, Cubaka VK, Makasa M, Guyse A, De Maeseneer J. A scoping review on family medicine in sub-Saharan Africa: practice, positioning and impact in African health care systems. Human Resources for Health. 2020;18(1):27. doi: 10.1186/s12960-020-0455-4 32245501 PMC7126134

[94] Montegut A, Schrmer J, Cartwright C, Holt C, Chuc NTK, An PN, et al. Creation of postgraduate training programs for family medicine in Vietnam. Family Medicine. 2007;39(9):634–8. 17932796

[95] Abyad A, Al-Baho AK, Unluoglu I, Tarawneh M, Al Hilfy TK. Development of family medicine in the middle East. Family Medcine. 2007;39(10):736–41. 17987417

[96] Evensen A, Wondimagegn D, Zemenfes Ashebir D, Rouleau K, Haq C, Ghavam-Rassoul A, et al. Family Medicine in Ethiopia: Lessons from a Global Collaboration. Journal of the American Board of Family Medicine. 2017;30(5):670–7 doi: 10.3122/jabfm.2017.05.170086 28923820

[97] Hayes B, Shrestha A. Historical evolution and present status of general practice in Nepal. Journal of General Practice and Emergency Medicne of Nepal. 2014;3(4).

[98] Ponka D, Rouleau K, Arya N, Redwood-Campbell L, Woollard R, Siedlecki B, et al. Developing the evidentiary basis for family medicine in the global context. Canadian Family Physician. 2015;61. PMC4501601 26380849

